# Antifungal mechanism of volatile compounds emitted by *Actinomycetota Paenarthrobacter ureafaciens* from a disease-suppressive soil on *Saccharomyces cerevisiae*

**DOI:** 10.1128/msphere.00324-23

**Published:** 2023-09-26

**Authors:** Tri-Phuong Nguyen, De-Rui Meng, Ching-Han Chang, Pei-Yu Su, Chieh-An Ou, Ping-Fu Hou, Huang-Mo Sung, Chang-Hung Chou, Masaru Ohme-Takagi, Hao-Jen Huang

**Affiliations:** 1 Department of Life Sciences, National Cheng Kung University, Tainan, Taiwan; 2 Graduate Program in Translational Agricultural Sciences, National Cheng Kung University and Academia Sinica, Tainan, Taiwan; 3 Kaohsiung District Agricultural Research and Extension Station, Pingtung, Taiwan; 4 Institute of Tropical Plant Sciences and Microbiology, National Cheng Kung University, Tainan, Taiwan; University of Georgia, Athens, Georgia, USA

**Keywords:** *Paenarthrobacter ureafaciens*, phytopathogens, antifungal mechanism, volatile organic compounds, volatile allelochemicals, allelopathy, microbial volatile compounds

## Abstract

Increasing evidence suggests that in disease-suppressive soils, microbial volatile compounds (mVCs) released from bacteria may inhibit the growth of plant-pathogenic fungi. However, the antifungal activities and molecular responses of fungi to different mVCs remain largely undescribed. In this study, we first evaluated the responses of pathogenic fungi to treatment with mVCs from *Paenarthrobacter ureafaciens*. Then, we utilized the well-characterized fungal model organism *Saccharomyces cerevisiae* to study the potential mechanistic effects of the mVCs. Our data showed that exposure to *P. ureafaciens* mVCs leads to reduced growth of several pathogenic fungi, and in yeast cells, mVC exposure prompts the accumulation of reactive oxygen species. Further experiments with *S. cerevisiae* deletion mutants indicated that Slt2/Mpk1 and Hog1 MAPKs play major roles in the yeast response to *P. ureafaciens* mVCs. Transcriptomic analysis revealed that exposure to mVCs was associated with 1,030 differentially expressed genes (DEGs) in yeast. According to gene ontology and Kyoto Encyclopedia of Genes and Genomes analyses, many of these DEGs are involved in mitochondrial dysfunction, cell integrity, mitophagy, cellular metabolism, and iron uptake. Genes encoding antimicrobial proteins were also significantly altered in the yeast after exposure to mVCs. These findings suggest that oxidative damage and mitochondrial dysfunction are major contributors to the fungal toxicity of mVCs. Furthermore, our data showed that cell wall, antioxidant, and antimicrobial defenses are induced in yeast exposed to mVCs. Thus, our findings expand upon previous research by delineating the transcriptional responses of the fungal model.

**IMPORTANCE:**

Since the use of bacteria-emitted volatile compounds in phytopathogen control is of considerable interest, it is important to understand the molecular mechanisms by which fungi may adapt to microbial volatile compounds (mVCs). *Paenarthrobacter ureafaciens* is an isolated bacterium from disease-suppressive soil that belongs to the *Actinomycetota* phylum. *P. ureafaciens* mVCs showed a potent antifungal effect on phytopathogens, which may contribute to disease suppression in soil. However, our knowledge about the antifungal mechanism of mVCs is limited. This study has proven that mVCs are toxic to fungi due to oxidative stress and mitochondrial dysfunction. To deal with mVC toxicity, antioxidants and physical defenses are required. Furthermore, iron uptake and CAP proteins are required for antimicrobial defense, which is necessary for fungi to deal with the thread from mVCs. This study provides essential foundational knowledge regarding the molecular responses of fungi to inhibitory mVCs.

## INTRODUCTION

The chemical communications between different organisms are collectively known as allelopathy (1). In one type of allelopathy, an organism may release toxic compounds into the surrounding medium, and the released allelochemicals may elicit toxicity and defense responses in another target organism (2, 3). Allelopathy is known to play important roles in agricultural practices such as weed control and crop re-establishment (4, 5). In soil, extensive allelopathic interactions occur between soil bacteria and fungi (6 – 8), and the inhibition of plant pathogens by allelochemicals produced by soilborne bacteria can indirectly contribute to plant health (9).

It is widely accepted that microbial volatile compounds (mVCs) can inhibit pathogenic fungi (10 – 13). The physiochemical characteristics of mVCs enable the compounds to quickly distribute throughout the network of soil pores and effectively interfere with pathogenic fungi (14 – 16). Exposures of different fungi to mVCs have been shown to trigger toxic effects such as oxidative stress and damage to the integrity of fungal cell walls (8, 17 – 19). As such, reactive oxygen species (ROS) scavengers are expected to play vital roles in protecting target cells from mVC-induced oxidative stress (17, 20). Moreover, the cellular levels and effects of ROS can be modulated by the activities of specific mitogen-activated protein kinase (MAPK) cascades (18, 21). MAPKs are pivotal components of intracellular signaling pathways that respond to environmental signals, control infection, and modulate the development of pathogenic fungi (22, 23). Thus, the initial states and responses of intracellular antioxidants and MAPK signaling pathways may greatly influence the effects of mVCs in bacterial-fungal interactions and competitive interactions.

Disease-suppressive soils are those in which host plants are well protected from pathogenic infections due to the antifungal activities of soil microorganisms (11, 24, 25). Of note, the disease-suppressive nature of most soils is attributed to the presence of diverse microbial communities, which have the potential to combat soilborne pathogens (26). Bacteria of the *Actinomycetota* phylum are one of the five most dominant reported in soils (27). Intriguingly, our previous study showed that *Actinomycetota* are major contributors to *Rhizoctonia solani* inhibition in soil (28), and from *Rhizoctonia*-suppressive soil, we isolated *Paenarthrobacter ureafaciens*, which belongs to the *Actinomycetota* phylum (29, 30). *P. ureafaciens* is known as an indole acetic acid and siderophore producer (31) as well as an herbicide-degrading bacterium (32, 33). Thus, *Actinomycetota P. ureafaciens* may impact plant growth and microbial communications in soil. However, it remains unknown if and how mVCs from *P. ureafaciens* suppress growth and cause toxicity in plant pathogens.

*Saccharomyces cerevisiae* is an especially useful model organism for studying antifungal mechanisms (21, 34), as this species has been extensively characterized in terms of its cellular signaling responses to internal and external stimuli (35). Although numerous fungal genomes have been completely sequenced, the cellular processes in budding yeast are the most completely elucidated (36). Moreover, the abundance of mutant strains and well-characterized protein-protein interactions facilitate rapid and precise targeting of many pathways in laboratory studies (37). The recent development of RNA sequencing (RNA-seq) has also enabled researchers to reveal extensive molecular changes that occur in yeast exposed to environmental stresses (38 – 41). As a method of inquiry, RNA-seq has considerable benefits for gene expression studies (42). Therefore, transcriptomic analysis of *S. cerevisiae* can offer a unique perspective on the molecular impacts of mVCs on pathogenic fungi.

The contributions of soil bacteria to plant health are a cornerstone of ecosystem function in disease-suppressive soils (9, 43, 44). The bacteria function in this role by acting as a seemingly inexhaustible source of mVCs to inhibit plant-pathogenic fungi (13, 16). In this study, we first sought to determine whether mVCs released by *P. ureafaciens* have the potential to inhibit the growth of phytopathogenic fungi. Then, we wanted to delineate the molecular modes of action involved in toxicity and fungal response to mVCs. To assess how fungi respond to mVCs and how toxicity occurs, we performed bioassays on the *S. cerevisiae* fungal model. Our findings reveal previously unknown transcriptional responses in fungi exposed to mVCs and implicate several key biomolecules in the mechanisms of toxicity and resistance.

## RESULTS

### *P. ureafaciens* mVCs inhibit phytopathogenic fungi and yeast growth

*P. ureafaciens* was first tested for its ability to produce mVCs that could inhibit the growth of phytopathogens. As expected, the volatile compounds from this bacterial species effectively reduced the growth of several pathogenic fungi, with inhibition percentages ranging from 46.04% to 73.41% (Fig. S2 and S3). For the soilborne pathogenic fungus, *R. solani*, growth was inhibited by 62.54% (Fig. S3) as compared with unexposed controls. For fruit pathogenic fungi, the most strongly inhibited strain was *Lasiodiplodia theobromae* (73.41% inhibition). Meanwhile, the growth inhibition of *Colletotrichum siamense*, *Colletotrichum gloeosporioides*, and *Neofusicoccum parvum* was 46.04%, 48.84%, and 52.62%, respectively (Fig. S3). Taken together, these data showed that *P. ureafaciens* mVCs can consistently inhibit the growth of different phytopathogenic fungi.

**FIG 1:**
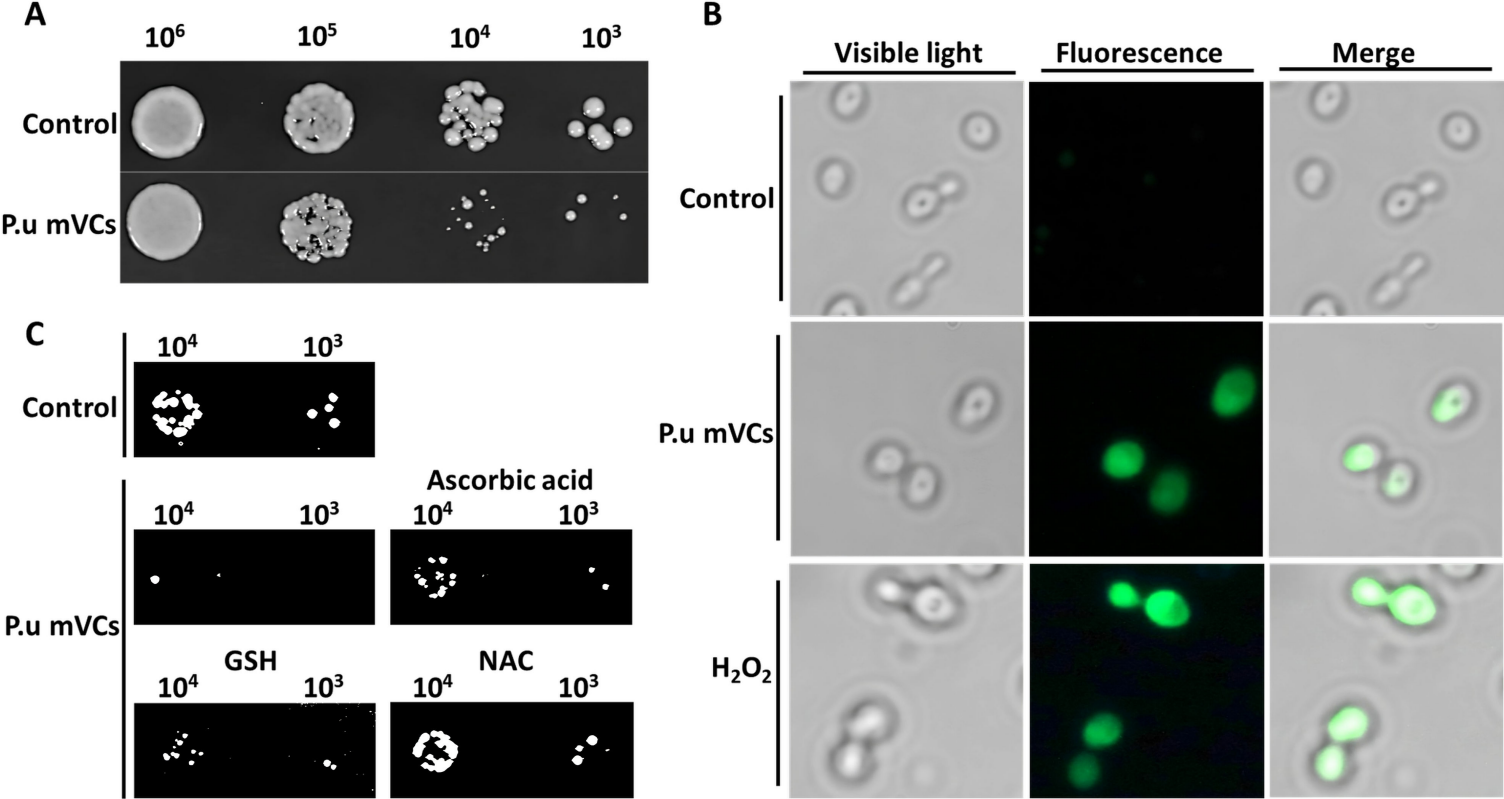
Antifungal effect of *P. ureafaciens* on yeast. (**A**) mVCs inhibit the growth of yeast cells. Tenfold dilutions of yeast were spotted on SD medium. (**B**) *P. ureafaciens* mVCs induced ROS accumulation in *S. cerevisiae* BY4741 cells. H_2_O_2_ treatment served as a positive control. (**C**) ROS scavenging effects in WT BY4741 exposed to mVCs of *P. ureafaciens* for 48 h. ROS scavengers (ascorbic acid, GSH, or NAC) were added to the SD medium. The ROS accumulation and cell viability were assessed with or without exposure to *P. ureafaciens* mVCs (P.u mVCs). The data are derived from three independent experiments.

Among all eukaryotic organisms, *S. cerevisiae* is one of the most extensively studied (45). Due to the vast abundance of available yeast mutants and their well-characterized signaling networks, we chose to use this species for our analysis of how mVCs affect the yeast fungal model in terms of their effects on specific pathways. To verify that our results obtained for phytopathogens correspond to the effects in yeast, we used two-section Petri dishes to expose the yeast to mVCs (Fig. S1B). At a density of 10^8^ CFU·mL^−1^ *P. ureafaciens*, the co-cultured yeast displayed high sensitivity to mVCs at densities from 10^5^ to 10^3^ and little inhibitory activity at a density of 10^6^ (Fig. 1A), suggesting that the *P. ureafaciens* mVCs may confer antagonistic effects on yeast, as was observed with other fungi.

### mVCs induce ROS accumulation in yeast cells

Excessive ROS accumulation leads to oxidative damage of lipids, proteins, and DNA (21). To test whether ROS accumulates in yeast exposed to *P. ureafaciens* mVCs, the 2′,7′-dichlorofluorescein diacetate (DCFH-DA) ROS sensor was used. *P. ureafaciens* mVCs induced significant ROS accumulation in *S. cerevisiae* at a density of 10^6^ (Fig. 1B). Next, we supplemented the media of wild-type (WT) BY4741 yeast cells with ascorbic acid, glutathione (GSH), or N-acetyl cysteine (NAC) ROS scavengers during the mVC treatment. The antioxidant supplements all safeguarded yeast cells against mVC-induced cytotoxicity at densities of 10^4^ and 10^3^ (Fig. 1C). Our results therefore suggest that oxidative stress may play a critical role in *P. ureafaciens* mVC-induced growth inhibition.

### Slt2/Mpk1 and Hog1 are critical factors controlling tolerance to mVC-mediated stress

**FIG 2:**
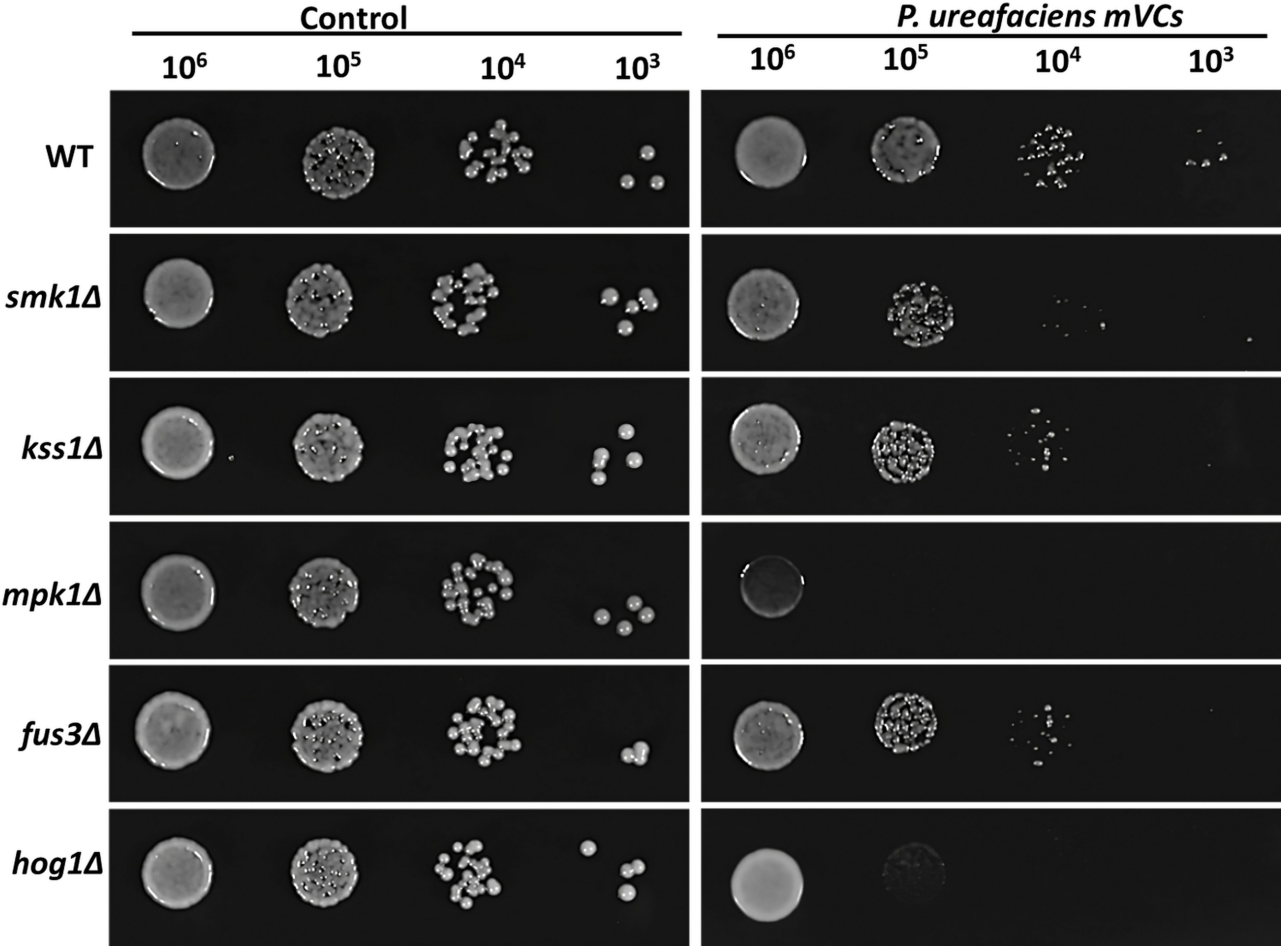
Deletion of mpk1 and hog1 sensitizes yeast to mVCs. The cell viabilities of WT and mutant strains (kss1Δ, fus3Δ, hog1Δ, smk1Δ, mpk1Δ) on BY4741 backgrounds were assessed with or without exposure to *P. ureafaciens* mVCs. The data are derived from three independent experiments.

To identify critical factors in the regulation of the mVC-induced stress response, we tested the importance of five MAPKs (Slt2/Mpk1, Hog1, Kss1, Fus3, and Smk1) in the response of *S. cerevisiae* to *P. ureafaciens* mVCs. The *mpk1*Δ strain showed the most significant decreases in cell viability at densities ranging from 10^6^ to 10^3^, and the *hog1*Δ strain completely inhibited the growth at densities from 10^5^ to 10^3^ in comparison to the WT BY4741 strain. However, no significant effects were observed at densities from 10^6^ to 10^4^ when comparing kss1, fus3, and smk1 mutants to the WT strain (Fig. 2). These results suggest that filamentous growth, pheromone, and sporulation signaling pathways are likely to only play minor roles in mVC resistance. Interestingly, both Mpk1/Slt2 and Hog1 are stimulated by oxidative stress (46, 47). Therefore, it is possible that crosstalk between these MAPK pathways may partly contribute to mVC-induced stress tolerance.

### The transcriptional stress response of *S. cerevisiae* and *P. ureafaciens* mVCs

**FIG 3:**
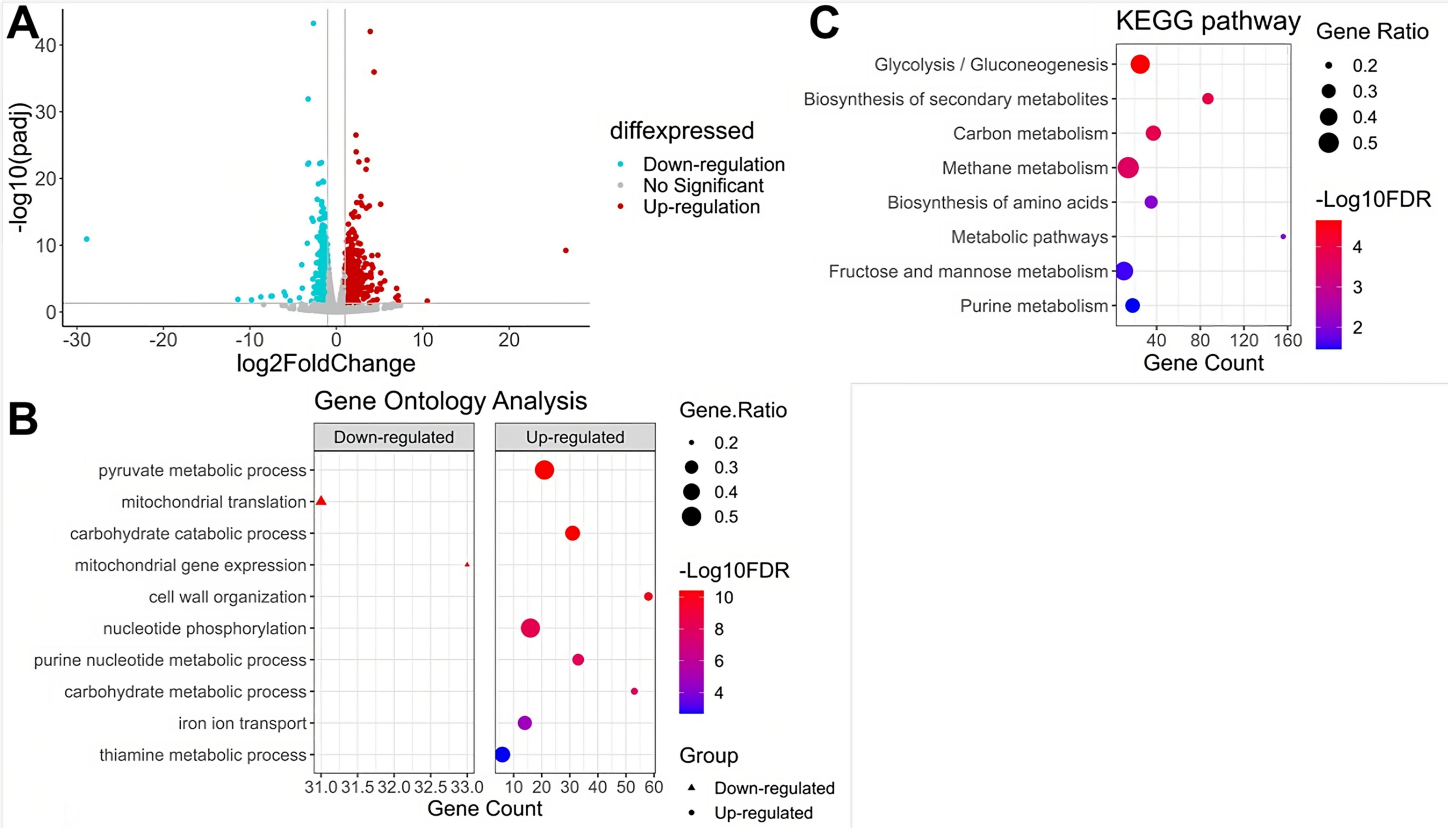
*P. ureafaciens* mVCs induce a transcriptional defense response in *S. cerevisiae*. (**A**) The volcano plots of the DEGs. Significant DEGs are shown as red dots (upregulated) or blue dots (downregulated); unaffected genes are shown as gray dots. The abscissa represents log2(fold change), and the ordinate represents statistical significance level. (**B**) GO of DEGs in *S. cerevisiae* after exposure to *P. ureafaciens* mVCs. (**C**) KEGG pathway enrichment analysis of DEGs. Red and blue colors represent higher and lower statistical significance, respectively. The bar represents the negative log of FDR (base 10). Black dots represent the ratios of genes annotated to categories. The abscissa represents gene counts, and the ordinate represents enrichment terms. The data are derived from three independent experiments.

To track alterations in the gene expression profile of *S. cerevisiae*, yeast cells were incubated with or without *P. ureafaciens* mVCs for 48 h and collected for RNA-Seq with Illumina NGS technology. Exposure to *P. ureafaciens* mVCs was associated with a total of 1,030 differentially expressed genes (DEGs) (Fig. 3A), including 634 upregulated genes (Table S1) and 396 downregulated genes (Table S2 and S3). To classify the DEGs, we performed gene ontology (GO) term analysis. Among the mVC-upregulated DEGs, enrichments were found for several terms, including ion transport, cell wall organization, and metabolic processes such as purine, carbohydrate, and vitamin (Fig. 3B). On the other hand, mitochondrial translation and mitochondrial gene expression were significantly enriched with mVC-downregulated DEGs (Fig. 3B). To further characterize the pathways regulated by *P. ureafaciens* mVCs, we then performed Kyoto Encyclopedia of Genes and Genomes (KEGG) pathway analysis. The DEGs were mainly involved in eight KEGG metabolic pathways, including glycolysis, secondary metabolites, carbon metabolism, amino acid biosynthesis, metabolic pathways, fructose and mannose metabolism, and purine metabolism (Fig. 3C).

### *P. ureafaciens* mVCs induce purine and thiamine biosynthesis

Purine is a crucial biomolecule that is necessary for DNA and RNA (adenine and guanine nucleotides) and major cellular cofactors (NAD and FAD) (48). In *S. cerevisiae*, purine biosynthesis is also involved in stress resistance (49). This pathway consists of a group of adenine-requiring (ADE) genes that generate inosine monophosphate, a branch point in the synthesis of adenine and guanine (48). Hence, we examined the related genes in our RNA-seq data to better understand how purine biosynthesis is affected by *P. ureafaciens* mVCs. Several genes involved in purine biosynthesis were highly expressed, including *ADE4*, *ADE5*,*7*, *ADE6*, *ADE2*, *ADE1*, *ADE12*, *IMD3*, *IMD4*, *RNR3*, *AAH1*, and *ADK1* (Fig. S2). Among these ADE genes, *ADE17* plays an especially essential role in promoting growth and fermentation under stress conditions (50, 51).

**FIG 4:**
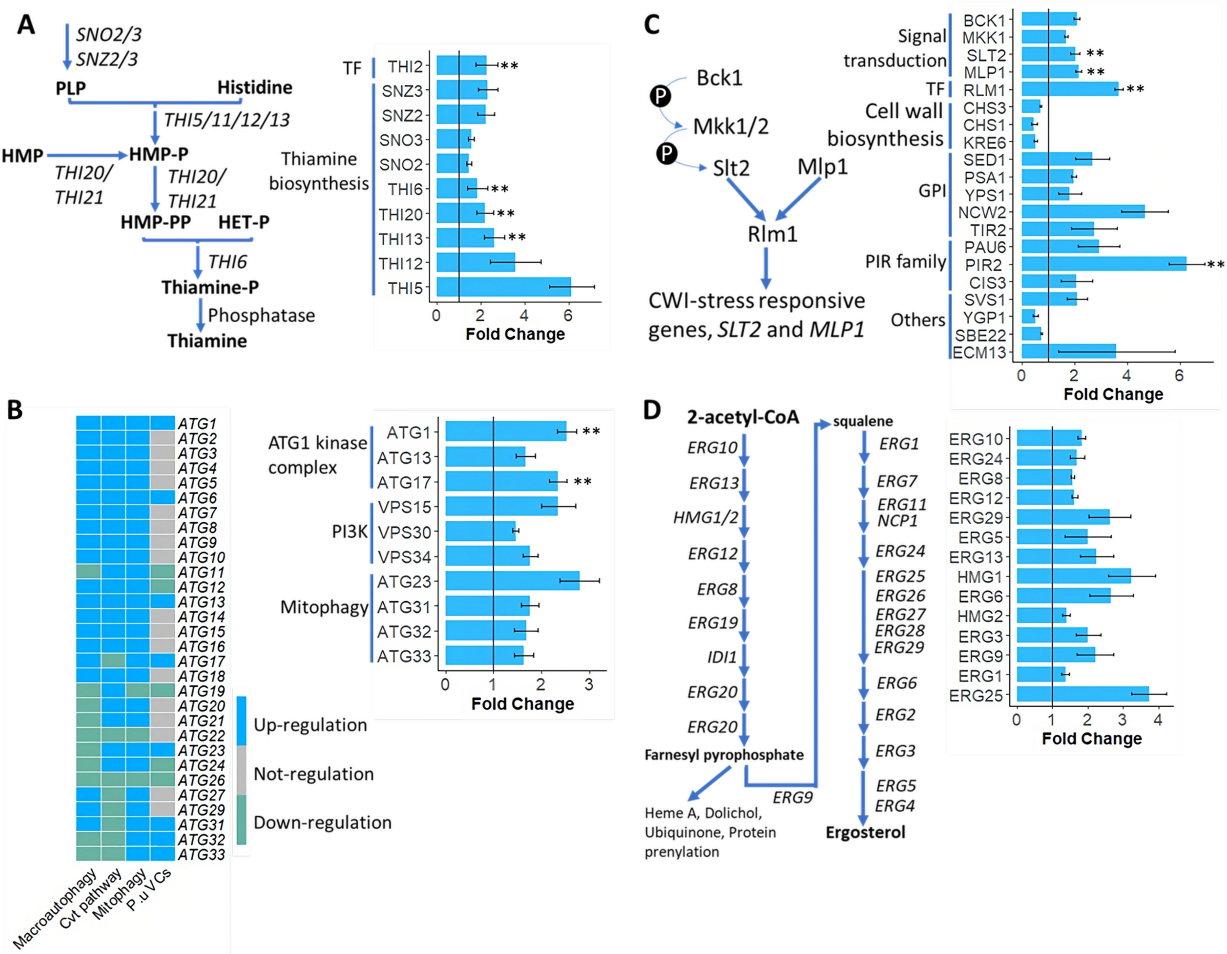
Exposure to *P. ureafaciens* mVCs triggers defensive responses in yeast. (**A**) The expression patterns of thiamine biosynthesis genes were significantly induced by exposure to mVCs. (**B**) Autophagy-related genes induced by mVC-triggered stress are likely to enhance mitophagy. Comparison of the expression patterns among macroautophagy, the Cvt pathway, mitophagy (54), and autophagy-related genes in response to *P. ureafaciens* mVCs (left). Up- and downregulation are indicated by blue and green, respectively; gray indicates no significant change. The autophagy-related genes were induced by exposure to *P. ureafaciens* mVCs (right). (**C**) Expression patterns of the Mpk1/Slt2 pathway and downstream CWI-related targets were altered by mVC-triggered stress. (**D**) ERG genes were induced by exposure to *P. ureafaciens* mVCs. Gene expression changes validated by qPCR are indicated by **. The abscissa represents the fold change in expression for each gene named on the ordinate. The data are derived from three independent experiments.

Thiamine not only serves as a cofactor for various enzymes but also stimulates yeast survival through the activation of thiamine-dependent stress protection mechanisms (52). To confirm that thiamine signaling was triggered by mVCs, we performed RT-qPCR to track the levels of *THI2*, a gene encoding a key transcriptional regulator of THI genes (53). *THI2* was significantly upregulated by 2.25-fold after treatment with mVCs (Fig. 4A), suggesting that downstream genes in the thiamine biosynthesis pathway should be activated. In addition, we investigated the expression levels of genes related to thiamine biosynthesis in our RNA-seq data, which allowed us to understand how the pathway responds to *P. ureafaciens* mVC exposure. Many genes involved in the thiamine metabolism, including *THI5,12,13,20* and *SNZ2,3*, showed significant upregulation of expression after the treatment (Fig. 4A).

### mVCs induce mitochondrial dysfunction and mitophagy in yeast

Exogenous stimuli like antifungal volatiles may cause mitochondrial dysfunction, which can lead to oxidative stress, decreased cell viability, and cell death (55, 56). As such, many antifungal compounds target mitochondria as a major mechanism of action (57). Therefore, we sought to assess the state of mitochondria in *S. cerevisiae* exposed to *P. ureafaciens* mVCs. We found downregulated expression of genes related to mitochondrial translation and mitochondrial gene expression after *P. ureafaciens* mVC treatment (Fig. 3B). This transcriptional regulation may contribute to the inhibitory mechanism of antifungal volatiles produced by *P. ureafaciens*.

Mitophagy plays a vital role in controlling mitochondrial quality and reducing oxidative stress (54, 58), and it is also critical for phytopathogen virulence (59, 60). Therefore, we investigated whether the *P. ureafaciens* mVCs might affect determinants of mitophagy in the yeast model system. Intriguingly, both *ATG32* and *ATG33* were significantly upregulated by mVC exposure (Fig. 4B). Moreover, the genes encoding the ATG1 kinase complex and yeast PI3K complex I were significantly induced by mVC treatment (Fig. 4B). By comparing the autophagy-related genes induced by *P. ureafaciens* mVCs to known genes in the macroautophagy and Cvt pathways (54), we determined that the expression patterns induced by *P. ureafaciens* mVCs are most likely to modulate mitophagy (Fig. 4B). Thus, we can further surmise that upregulation of mitophagy is likely to safeguard yeast cells against oxidative damage induced by mVCs.

### mVCs alter the expression of the cell wall integrity pathway and ergosterol genes

Yeast triggers the well-characterized cell wall integrity (CWI) pathway as a response to cell wall stress from certain environmental stimuli (61, 62). In our data set, several genes encoding cell wall components and cell wall synthesis factors were transcriptionally affected by mVC exposure (Fig. 4C). *BCK1*, *MKK1*, *SLT2*, and *MLP1* each encode signal transduction components of the CWI pathway, and *RLM1* encodes the MADS-box transcription factor. All of these genes were upregulated upon mVC treatment (Fig. 4C). In addition, genes encoding glycosyl-phosphatidylinositol (GPI) proteins (i.e., *SED1*, *PSA1*, *YPS1*, *NCW2*, *TIR2*) and members of the PIR family (i.e., *PAU6*, *PIR2*, *CIS3*) were similarly upregulated (Fig. 4C). In contrast, genes involved in chitin synthesis (*CHS1*, *CHS3*) and 1–6 β-glucan synthesis (*KRE6*) were significantly downregulated. Most of those genes are known to be Slt2-dependent (63, 64), suggesting that the MAPK-CWI pathway was activated in response to mVC-triggered stress.

Together with the cell wall, the outer cell membrane is one of the first defensive structures encountered by environmental stressors (65). Ergosterol (ERG) is an important component of biological membranes in fungi (66), so we also examined the expression patterns of ergosterol synthesis genes in response to *P. ureafaciens* mVC exposure. We found that ERG biosynthesis genes, including *HMG1* (encoding for HMG-CoA reductase), *ERG3*, *ERG5*, *ERG6*, *ERG9*, *ERG13*, *ERG25*, and *ERG29*, were all substantially upregulated by mVC exposure (Fig. 4D). The significant expression increases in ERG-related genes suggest that cell membrane integrity is a key defensive factor in yeast cells challenged with mVCs.

### Volatile compounds produced by *P. ureafaciens* induce antimicrobial defense in yeast

**FIG 5:**
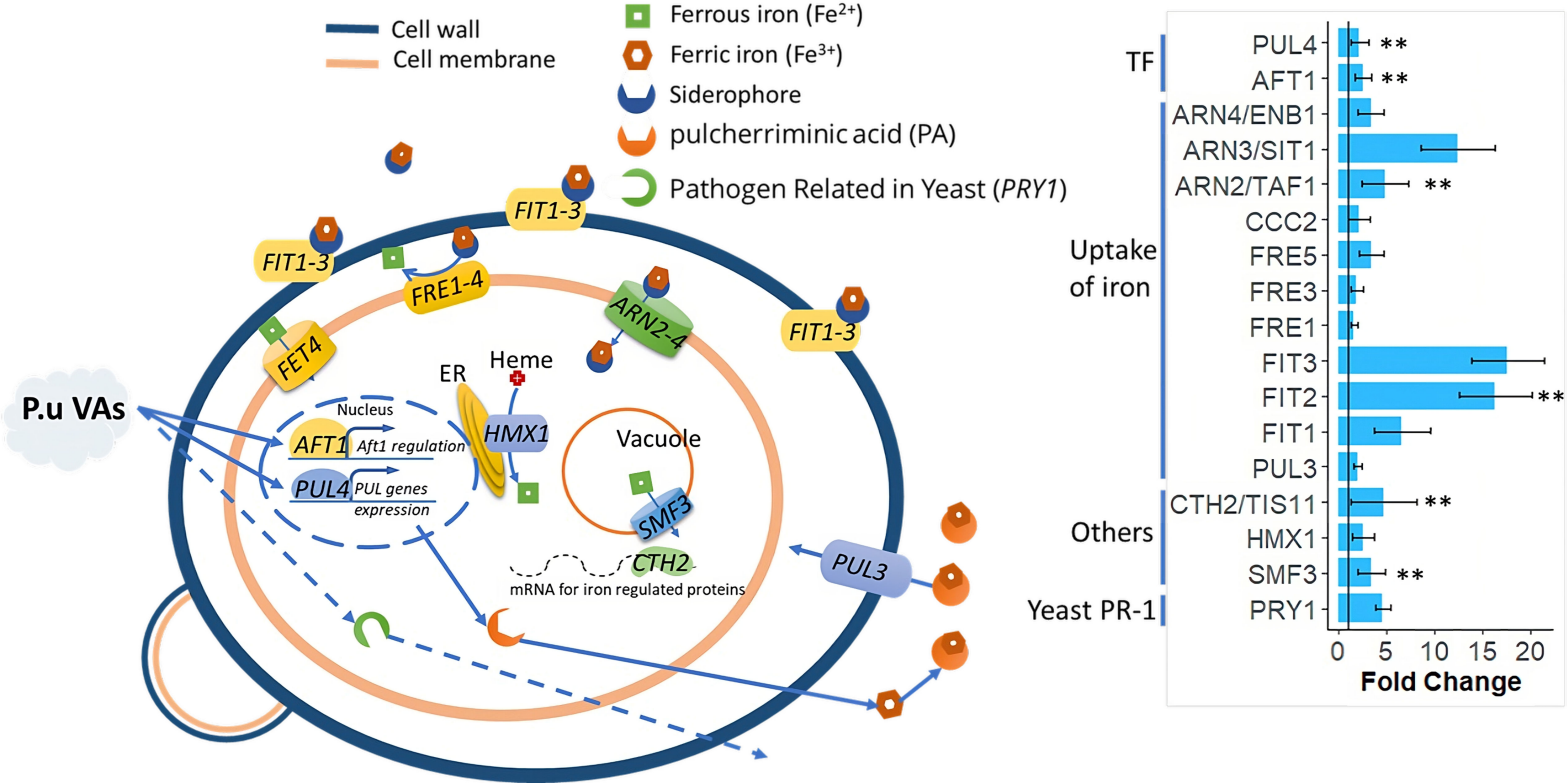
Exposure to mVCs induces antimicrobial defense in yeast. Genes with expression significantly affected by *P. ureafaciens* exposure (fold change >2; adjusted *P* < 0.05) compared to controls are shown in the pathway (left). Gene expression levels are shown in the chart (right). Validated genes by qPCR were indicated by **. The abscissa represents fold changes, and the ordinate represents gene names. The data are derived from three independent experiments.

Iron is an essential nutrient that is required for the activities of numerous enzymes involved in a wide variety of cellular processes (67). In yeast, Aft1 transcription factors are critical for the organism to mount a response to iron depletion (68). We observed transcriptional upregulation of *AFT1*, an iron uptake activator (68), and siderophore transport components *FIT1-3* and *ARN2-4* (67, 69, 70) (Fig. 5). Additionally, we saw upregulation of *PUL4* (the iron-transporting pulcherrimin transcription factor) and *PUL3* (the pulcherrimin transporter) (71) (Fig. 5). This result suggests that the iron uptake pathway may play an important role in protecting yeast from mVC exposures. Along with the iron uptake pathway, *PRY1* expression was induced by *P. ureafaciens* mVC exposure (Fig. 5). This gene encodes a pathogen-related protein in yeast, Pry1, which belongs to the fatty acid-binding CAP protein superfamily (72). In plants, the activity of PR-1 proteins has been shown to combat pathogen infections (73). The expression of Pry1 in yeast may be similarly related to antimicrobial defense, though this idea has not been experimentally tested.

## DISCUSSION

In this study, we first evaluated the antifungal effects of mVCs from *P. ureafaciens* on the plant pathogen *R. solani*. The growth of this pathogen was significantly inhibited by *P. ureafaciens* mVCs (Fig. S3). In addition to the presence of the soilborne pathogen *R. solani*, fruit pathogenic fungi may inhabit the soil and become integrated into the terrestrial microbial community (25). Consequently, these fruit pathogens can extensively interact with other microbial soil inhabitants. Thus, we also examined the antifungal effects of *P. ureafaciens* mVCs on fruit pathogens, including *C. gloeosporioides*, *L. theobromae*, *N. parvum*, and *C. siamense*. Exposure to mVCs effectively reduced the growth of all tested pathogenic fungi (Fig. S3). Taken together, our data showed that the mVCs can suppress a wide range of phytopathogenic fungi *in vitro* and may act as antifungal compounds. Importantly, while the intricacy of the soil environment poses challenges to the production and activity of mVCs, mVCs produced by pure cultures can also suppress phytopathogens in soil atmospheres (13), suggesting that the emitted molecules may contribute to soil fungistasis. Since the use of bacteria-emitted mVCs in biological control of agricultural settings has become a topic of considerable interest in recent years (74), it is important to understand the molecular mechanisms by which fungi may adapt to mVC exposure.

Yeast is thought to be an exceptional model organism for investigating the antifungal mechanisms of mVCs (21). Similar to the observed effects of mVCs on pathogens, yeast were also inhibited by the antifungal activity of *P. ureafaciens* mVCs (Fig. 1A). As mentioned earlier, mVCs often trigger ROS accumulation and cell wall damage in pathogenic fungi (8, 18). In line with this idea, we observed ROS accumulation (Fig. 1B) and activation of the CWI pathway (Fig. 3B and 4C) in yeast exposed to mVCs. Notably, the architecture, mechanisms, and physiological responses of numerous MAP kinase signaling cascades have been meticulously delineated in yeast (35), and the Mpk1/Slt2 MAPK pathway is known to be highly conserved and crucial for stress response in plant pathogenic fungi (75). Hence, the antifungal mechanisms we observed in yeast may be present in pathogenic fungi as well, and we expect that our use of yeast to explore the molecular effects of mVCs can provide novel and relevant insights into the antifungal activity of bacterial volatiles.

We found that exposure of yeast to mVCs induced ROS accumulation and negatively affected mitochondria (Fig. 1B and 3B; Table S2). These results were in line with our previous study, which showed that bacterial volatiles induce oxidative stress in yeast cells to potentially impact growth (21). Exogenous stimuli may negatively affect mitochondrial function to cause bursts of ROS production (76 – 78), and exposure to high levels of ROS will inevitably lead to damage to mitochondrial DNA and mitochondrial membranes (79). In this way, a negative feedback loop may be established, which could be crucial for the toxicity triggered by mVCs. As mentioned earlier, antioxidant defense systems are expected to play vital roles in safeguarding yeast cells from mVC-induced oxidative stress (17, 20). As such, although there was no significant increase [log2(fold change) ≥1 and adjusted *P* < 0.05] in enzymatic antioxidant genes such as catalase, superoxide dismutase, and peroxidase, we observed that non-enzymatic scavengers such as ascorbic acid, GSH, and NAC play an important role in cell survival under mVC-induced oxidative stress (Fig. 1C).

Mitophagy refers to the autophagy-dependent degradation of mitochondria, which serves as the primary mechanism for eliminating dysfunctional, aged, or excess mitochondria (54). Since this process plays a vital role in controlling mitochondrial quality and reducing oxidative stress (54, 58), we tested whether *P. ureafaciens* mVCs might trigger mitophagy in the yeast model. Indeed, we observed that mitophagy-related genes were induced upon exposure to *P. ureafaciens* mVCs (Fig. 4B). The Atg1-Atg13-Atg17 complex is thought to play a unique role in initiating autophagic machinery (80, 81), and it activates the phosphatidylinositol 3-kinase (PI3K) complex (82, 83), which is essential for phagophore formation (84 – 86). Of note, the mitochondrial outer membrane receptor Atg32 (87) is essential for the recruitment of mitochondria to the phagophore assembly site (PAS) (88, 89), and Atg33 contributes to the recruitment of aged or dysfunctional mitochondria by the PAS (90, 91). Interestingly, both Atg32 and Atg33 function in mitophagy but do not participate in other types of autophagy (54). Since oxidative stress-triggered mitophagy is responsible for eliminating dysfunctional mitochondria (92), proper regulation of oxidative stress and mitophagy is indispensable for cell survival and homeostasis (93). Intriguingly, our data suggest that the induction of mitophagy may be one mechanism of antioxidant defense that is initiated in response to mVC exposure.

Yeast possesses extensive defense systems to efficiently eliminate ROS and ensure the survival of cells under oxidative conditions. For instance, thiamine (vitamin B1) plays a vital role in safeguarding yeast from oxidative stress (52, 94). In this study, we noted that exposure to mVCs leads to upregulation of genes related to thiamine synthesis (Fig. 4A). Previous studies suggested that increases in the expression of thiamine biosynthesis genes allow yeast cells to survive oxidative stress in several conditions (94, 95). In addition to the effects on thiamine synthesis, genes involved in purine and histidine metabolism were induced by exposure to *P. ureafaciens* mVCs (Fig. S2), and the *BAS1* transcription factor (49) was highly expressed (Fig. S2). Purine metabolism has a major influence on the growth and development of fungi (96, 97), and *de novo* purine synthesis contributes to the detoxification of intracellular ROS (51). Therefore, the upregulation of thiamine and *de novo* purine biosynthesis pathways may represent important antioxidant defenses against the antifungal activity of mVCs.

In *S. cerevisiae*, MAPKs such as Fus3, Kss1, Hog1, Mpk1/Slt2, and Smk1 respond to external cues and mediate diverse cellular activities, including cellular fluctuating requirements, fusion, filamentous growth, osmotic imbalance, CWI, and meiosis (35, 98). In the present state of stress, Hog1 relates to cell cycle arrest, which is required to allow cells to generate adaptive responses before progressing into the next phase of the cycle (99). Furthermore, Mpk1/Slt2 is a tyrosine-1 (Tyr1) kinase and is involved in responding to DNA-damaging agents like hydroxyurea and phleomycin, as well as cooperating with TORC1 signaling in the presence of rapamycin (100). Among the mutants we tested, the mpk1Δ strain displayed the highest sensitivity in terms of cell viability (Fig. 2). Using qPCR, we also observed the significant expression of *MPK1/SLT2* (Fig. 4C), which then could activate the transcription factor Rlm1 to promote the expression of cell wall genes (62). In addition, the activation of the MAPK Hog1 is triggered by both cell wall stress (63) and oxidative stress (46), which leads to the subsequent activation of Rlm1 (101). In our experiments, cells lacking Hog1 showed better survival than those lacking Mpk1/Slt2 but worse survival than yeast without Kss1, Fus3, or Smk1 after exposure to mVCs (Fig. 2). Oxidative stress can induce cell wall stress in yeast (102) and activate both MAPKs Mpk1 and Hog1 (46, 47), which subsequently activate the Rlm1 transcription factor and induce genes related to CWI (62, 101). Therefore, we conclude that the MAPKs Mpk1/Slt2 and Hog1 may play important roles in protecting yeast cells from mVC-induced oxidative stress.

The CWI pathway in pathogenic fungi is essential for pathogenicity (103) and protection from external stresses (104, 105). Here, we observed that mVC exposure caused considerable changes in several cell wall-related genes, including GPI, the PIR family, glucan, and chitin synthesis/location (Fig. 4C). These CWI genes can be controlled by MAPKs Mpk1/Slt2 and Hog1 via the Rlm1 transcription factor (63, 64, 106). Interestingly, unlike the response to acetic acid stress (107), chitin and 1–6 β-glucan synthesis genes were negatively regulated in response to mVCs (Fig. 4C). On the other hand, ergosterol promotes plasma membrane integrity, permeability, and fluidity (108, 109). Owing to its crucial functions, many available antifungal agents disrupt the ergosterol biosynthesis pathway (108, 110). In our data, ERG biosynthesis genes were significantly upregulated after exposure of yeast to *P. ureafaciens* mVCs (Fig. 4D). Collectively, our findings suggest that the CWI pathway and ergosterol biosynthesis were activated by mVCs to promote the physical defenses of yeast cells. It is known that the expression of *S. cerevisiae* ERG genes can be controlled by the Hap1 heme-binding protein (111). Under conditions of iron deficiency, Hap1 switches from an activator to a repressor of ERG genes (111, 112). In light of this mechanism, it is likely that iron uptake may be critical for the yeast response to mVCs.

Pathogenic fungi have evolved efficient mechanisms for iron uptake to deal with iron scarcity in host tissues (113 – 115). On the other hand, siderophores from plant-protecting bacteria may lock iron away from pathogens to aid in pathogen suppression (116, 117). Therefore, competition for iron is a key factor in fungi-rhizobacteria interactions, and disruption of iron regulation may serve as a mechanism of antifungal activity. Although *S. cerevisiae* does not secrete siderophores, it can take up siderophore-bound iron excreted by other microorganisms (118, 119). We observed transcriptional upregulation of the iron uptake pathway, which suggested that it may help protect yeast from mVCs (Fig. 5). Siderophores are iron-chelating molecules, with many of them being non-ribosomal peptides that microorganisms may utilize to take up iron from the environment (115). These molecules can sequester iron, preventing uptake by microorganisms lacking appropriate receptors and effectively preserving the available iron for siderophore producers that possess cognate receptors. Consequently, organisms that are unable to compete by sequestering iron with their own siderophores experience severe iron deficiencies (120). Thus, siderophore production and iron uptake are likely to impact the antimicrobial defense capability of fungi.

In plants, PR-1 is a member of the CAP protein family (cysteine-rich secretory protein, antigen 5, a pathogenesis-related 1) and a key factor protecting against pathogens (121). Both yeast CAP proteins (Pry1, Pry2) and PR-1 proteins in plants possess the capability to bind to cholesteryl acetate within the secretory pathway and facilitate its export (72, 122). Our results showed upregulated expression of *PRY1* in yeast exposed to mVCs (Fig. 5). The PR-1 proteins cause harm to pathogens by allowing the plant to directly acquire sterols from the pathogen, which compromises its ability to infect the host (123). While it is possible that the induction of antimicrobial defense genes such as *PRY1* may help yeast defend against inhibitors, further examination will be required to determine the precise functions of these genes in yeast.

Collectively, our results reveal that mVCs emitted by *P. ureafaciens* act as antifungal agents, which can inhibit the growth of phytopathogenic fungi. Using the fungal model *S. cerevisiae*, we were able to suggest a plausible antifungal mechanism of *P. ureafaciens* mVCs. As such, our data showed that exposure to *P. ureafaciens* mVCs induces ROS accumulation and mitochondrial dysfunction in the yeast fungal model. Antioxidant defense systems, including thiamine, purine, and ROS scavengers, safeguard yeast cells against the toxicity of ROS induced by mVCs. We also indicated that the Mpk1/Slt2 and Hog1 pathways may play major roles in protecting yeast cell wall damage induced by mVC-induced oxidative stress. In particular, our data suggest that iron uptake and CAP proteins may play key roles in the defense response of yeast to mVCs from soilborne bacteria. However, the roles of these pathways in filamentous fungi are still not well defined (124, 125). Further studies will be needed to examine the suggested roles of iron uptake and CAP proteins in bacterial-fungal interactions. Our findings provide new insights into the antifungal mechanism of mVCs, but further research is required to determine the effects of individual mVCs from *P. ureafaciens* on mVC-induced stress. Moreover, further work will be required to validate the effects of mVCs on plant health in the presence of pathogens in greenhouse conditions.

## MATERIALS AND METHODS

### Strains, media, and growth conditions

The soil pathogen *Rhizoctonia solani* was obtained from the Food Industry Research and Development Institute, Taiwan. Fruit pathogens, *Colletotrichum gloeosporioides*, *Lasiodiplodia theobromae*, *Neofusicoccum parvum*, and *Colletotrichum siamense*, were isolated from infected fruits (respective BCRC numbers: FU31655, FU31653, FU31651, and FU31652).

The *S. cerevisiae* haploid BY4741 strain and corresponding deletion mutants were supplied by Rousseau and Bertolotti (126). The yeast was grown in yeast extract-peptone-dextrose (YPD) medium for routine maintenance. To conduct additional experiments, yeast cells were cultured at 30°C in synthetic defined (SD) medium (2% dextrose, 0.668% yeast nitrogen base without amino acids [Sigma]) plus amino acids (21). *Paenarthrobacter ureafaciens* is a soilborne bacterium (BCRC number 81269) isolated from a disease-suppressive soil in Kaohsiung District Agriculture Research and Extension Station, Pingtung, Taiwan. To measure the density of cell suspensions, a Hitachi U-2800A spectrophotometer (Hitachi Technologies, Japan) was used to measure absorbance at 600 nm (A600).

### Antifungal assay

To estimate the antifungal activity of mVCs released by *P. ureafaciens* on plant pathogenic fungi, a double-dish system was used (Fig. S1A). In this system, *P. ureafaciens* (10^8^ CFU·mL^−1^) was cultured in lysogeny broth (LB) medium at 28°C for 24 h. A mycelia agar plug (8 mm diameter) was positioned in the center of a potato dextrose agar (PDA) plate, which was then placed on top of the *P. ureafaciens*-containing plate. This “sandwich plate” culture was then sealed with parafilm and incubated at 28°C for 3 days. The sandwich plate arrangement only allows gas exchange between the fungus and bacterium chambers, not the outside environment. The control group was cultured under the same conditions but without *P. ureafaciens*. Fungal diameter was measured using ImageJ and quantified as percentage growth inhibition, according to the formula described by Ebadzadsahrai et al. (127).

To test the inhibitory effects of *P. ureafaciens* on *S. cerevisiae*, two-section Petri dishes with a physical barrier were used (Fig. S1B). First, *P. ureafaciens* (10^8^ CFU·mL^−1^) was cultured in LB medium at 28°C for 24 h. Then, the exponential phase of yeast cells was diluted to 10^6^ CFU·mL^−1^ and subsequently subjected to 10-fold serial dilutions. Next, 3 µL of each dilution was spotted on SD medium, and growth was assessed following a 48-h incubation period at 30°C.

### Evaluation of intracellular ROS content

To measure intracellular ROS levels, DCFH-DA (Sigma Aldrich D6883) was used. Budding yeast BY4741 cells at a density of 10^6^ were collected after treatment with or without *P. ureafaciens* mVCs and adjusted to an OD_600_ of 1.0. Then, the cells were centrifuged at 8,000 rpm for 1 min, and the supernatants were discarded. DCFH-DA (5 µM; diluted in PBS buffer containing 1 M K_2_HPO_4_ and 1 M KH_2_PO_4_) was added to resuspend the pellet. Then, the sample was shaken at 150 rpm at 28°C for 30 min in the dark. The ROS level was observed by visualizing yeast cells under fluorescence microscopy (Leica DMLB). A group treated with H_2_O_2_ was designated as the positive control.

### RNA extraction and sequencing

Yeast cells at a density of 10^6^ were incubated with *P. ureafaciens* (10^8^ CFU·mL^−1^) in two-section Petri dishes (Fig. S1B). After 48 h of exposure to *P. ureafaciens* mVCs, yeast cells were collected for total RNA extraction. Total RNA from yeast cells was extracted using the RNeasy Plant Mini kit (Qiagen, Hilden, Germany) and treated with DNase I (Roche, Basel, Switzerland). The RNeasy MInElute Cleanup Kit (Qiagen, Hilden, Germany) was used to purify RNA samples before quantification with a NanoDropTM 200 c Spectrophotometer (Thermo Scientific). An RNA-seq library was prepared from samples of total RNA extracted from *S. cerevisiae* with or without exposure to *P. ureafaciens* mVCs. The library was assembled using the Illumina NovaSeq platform, which generates 150 bp paired-end reads. Genewiz, Inc. (Plainfield, NJ, USA) performed the Illumina sequencing on three biological replicates.

Trimmomatic v0.36 was used to eliminate reads containing adaptor sequences and low-quality reads, with the quality score threshold set at 30 (128). Clean reads were aligned to the yeast genome (SGD) using TopHat v2.1.1. Then, Cufflinks v2.2.1 was used to calculate the abundance of transcript assemblies in fragments per kilobase of exon per million fragments mapped (129). The gene expression levels and DEGs were identified with Cuffdiff v2.2.1 (129). DEGs were identified using a threshold of |log2(fold change)| ≥1 and adjusted *P* < 0.05 as the criteria for a significant difference.

GO enrichment analysis of DEGs was performed using the enrichGO function of the clusterProfiler package (130). GO terms with corrected *P* < 0.05 were judged to be considerably enriched with DEGs. REVIGO was used to reduce redundant terms. To examine the enrichment of DEGs in the KEGG pathways, the gprofiler2 package was used for pathway mapping analysis (131). The Benjamini and Hochberg method was used to evaluate the false discovery rate (FDR). The significance threshold for each pathway was set at FDR <0.05.

### Quantitative RT-PCR validation

The expression levels of selected DEGs and antioxidant genes were measured by qRT-PCR. For each sample, 1,000 ng of RNA extracted from yeast cells was reverse transcribed using ImProm-IITM Reverse Transcriptase (Promega). The cDNA was subjected to qRT-PCR using PCR GoTaq qPCR Master Mix (Promega), and the RT-PCR was performed on a StepOnePlus Real-time PCR System (Applied Biosystems). The gene-specific primer sequences are listed in Table S3. In all experiments, Rdn18 served as an internal reference. The formula described by Livak and Schmittgen (132) was utilized to determine relative gene expression (132).

### Statistical analysis

All experiments were carried out with three independent replicates. Values are presented as mean ± SD. The statistical significance of each difference was assessed using Student’s *t*-test. *P* < 0.05 indicated significant differences.

## Data Availability

The RNA-seq data discussed in this study are currently available in NCBI’s Gene Expression Omnibus and are accessible through GEO (https://www.ncbi.nlm.nih.gov/geo/) with accession no. GSE240052.

## SUPPLEMENTAL MATERIAL

Supplemental Figures msphere.00324-23-s0001.docx Figures S1 to S3.

Supplemental tables msphere.00324-23-s0002.xlsx Tables S1 to S3.

ASM does not own the copyrights to Supplemental Material that may be linked to, or accessed through, an article. The authors have granted ASM a non-exclusive, world-wide license to publish the Supplemental Material files. Please contact the corresponding author directly for reuse.
