# Supplementary material for: Antifungal mechanism of volatile compounds emitted by Actinomycetota Paenarthrobacter ureafaciens from a disease-suppressive soil on Saccharomyces cerevisiae
Source: mSphere. 2023 Sep 26;8(5):e00324-23. doi: 10.1128/msphere.00324-23 (PMC10597458; doi:10.1128/msphere.00324-23)
Supplement: Supplemental Figures — Figures S1 to S3. [file msphere.00324-23-s0001.docx]

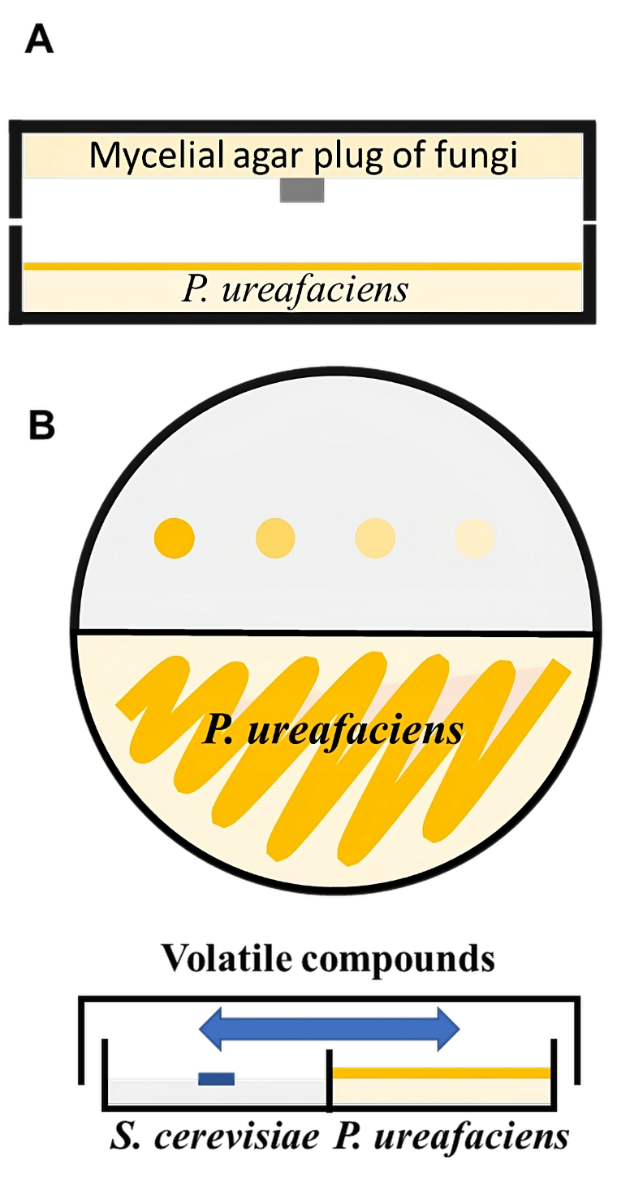


**Supplementary Fig. S1.** co-cultivation system. **A**) Sectional view of dual culture system; **B**) Co-cultivation system for yeast and *P. ureafaciens* in split Petri dish. Yeast incubated without *P. ureafaciens* was served as a control.


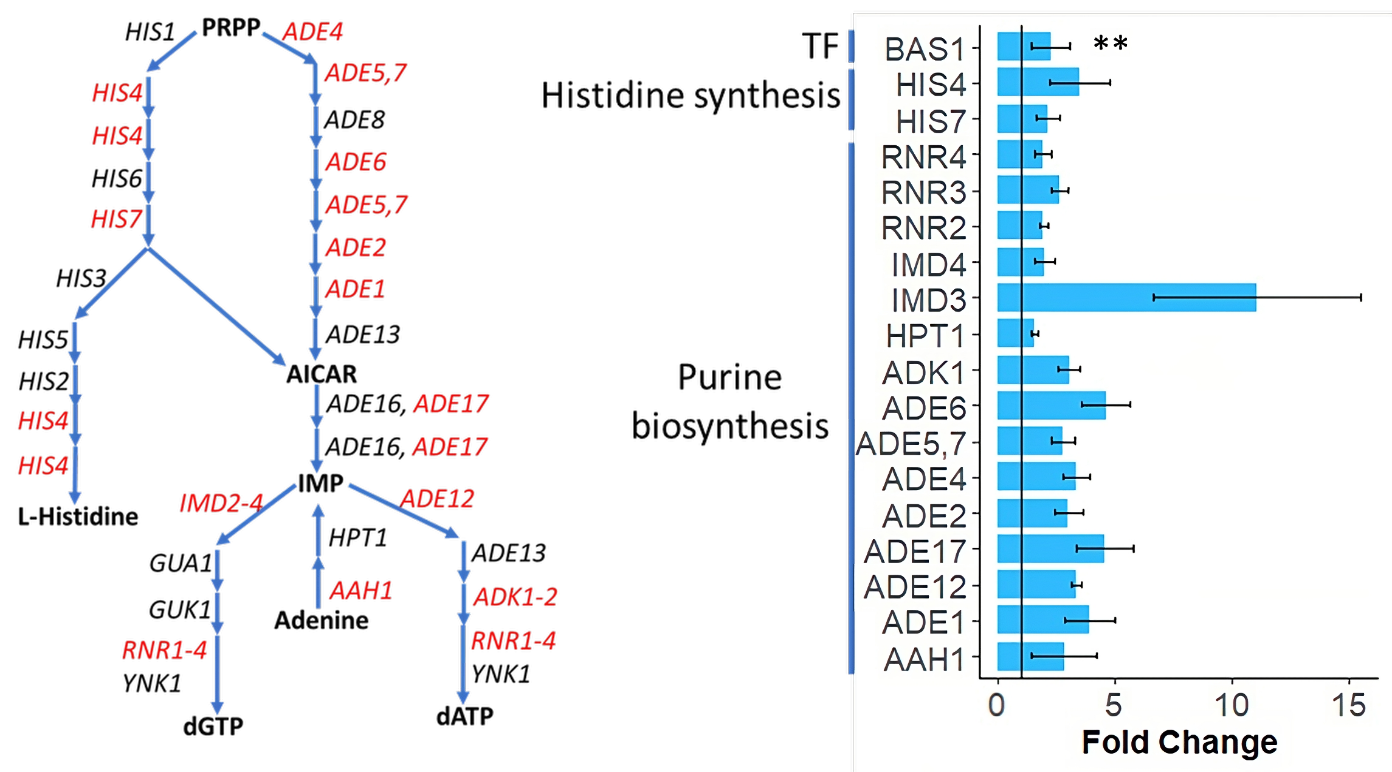


**Supplementary Fig. S2.** Exposure to *P. ureafaciens* mVCs triggers expressions of several genes in purine and histidine biosynthesis pathways. Gene name in red color represents for a significant change in expression (fold-change ≥ 2; p ≤ 0.05) compared to controls. Validated genes by qPCR were indicated by (**).


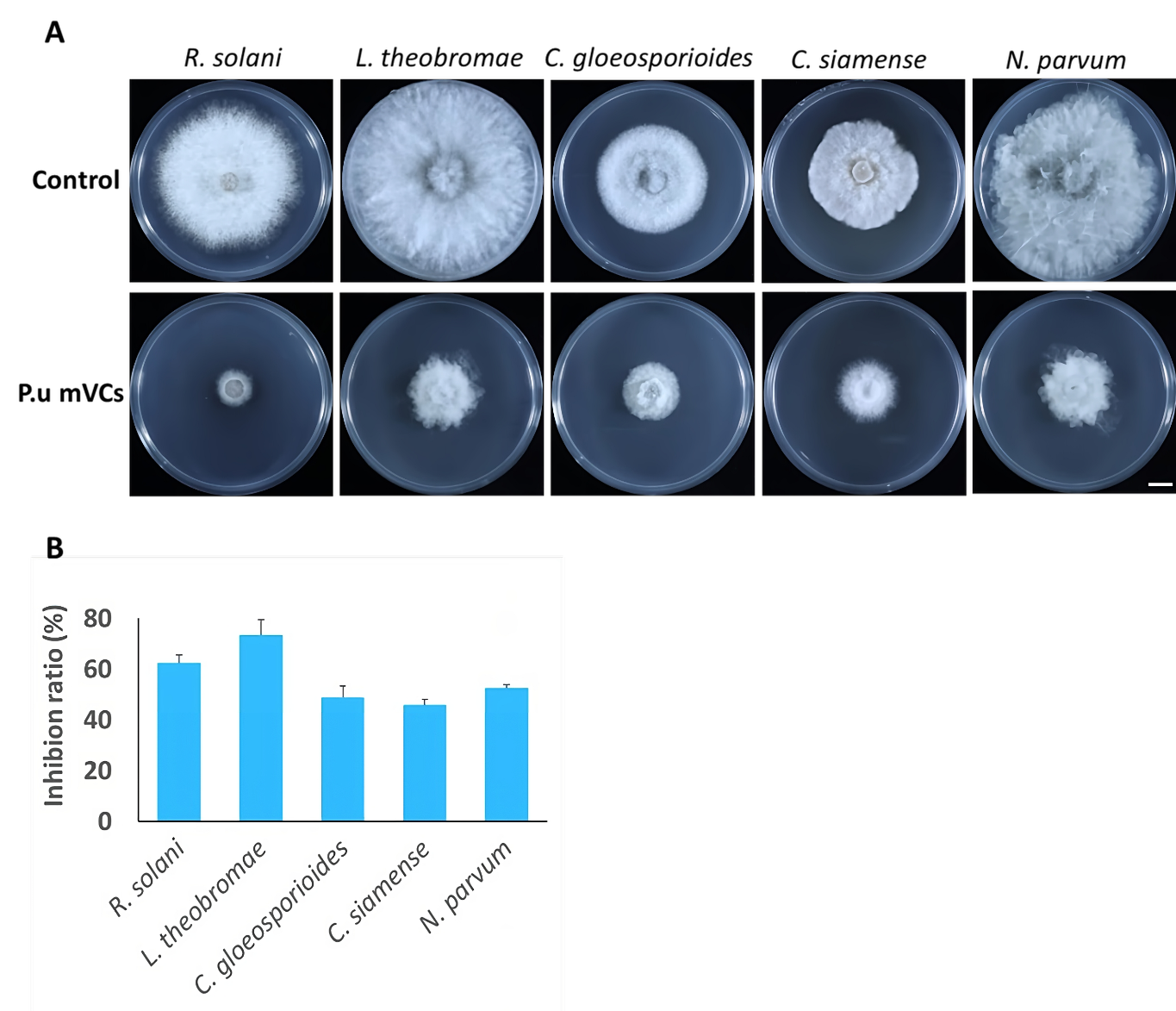


**Supplementary Fig. S3.** Antifungal effects of *P. ureafaciens* mVCs. **A)** mVCs produced by *P. ureafaciens* (P.u mVCs) inhibit growth of several species of phytopathogenic fungi (white bar, 1 cm). **B)** Growth inhibition of pathogenic fungi after exposure to mVCs (mean ± SD, n = 9). Colony diameter was evaluated from photos with ImageJ.
